# Correction: Fan et al. First Evidence of CpGV Resistance of Codling Moth in the USA. *Insects* 2022, *13*, 533

**DOI:** 10.3390/insects14010015

**Published:** 2022-12-23

**Authors:** Jiangbin Fan, Johannes A. Jehle, Ann Rucker, Anne L. Nielsen

**Affiliations:** 1 Key Laboratory of National Forestry and Grassland Administration on Management of Forest Bio-Disaster, College of Forestry, Northwest A&F University, Xianyang 712100, China; 2 Department of Entomology, Rutgers, The State University of New Jersey, Bridgeton, NJ 08302, USA; 3 Institute for Biological Control, Julius Kühn Institute (JKI)–Federal Research Centre for Cultivated Plants, 69221 Dossenheim, Germany

## Error in Figure

In the original publication [1], there was a mistake in Figure 1 and Figure 2 as published. The colors in the original bar graphs of the aforementioned Figure 1 and Figure 2 had disappeared in the first published version and uncolored graphs were released on the website. Consequently, those uncolored bar graphs made it inconvenient for readers to understand the bioassay results. There was a misspelled “cycle” in the Figure caption for Figure 1 and Figure 2. The corrected Figure 1 and Figure 2, and the proper spelling “circle” appear below. The authors state that the scientific conclusions are unaffected. This correction was approved by the Academic Editor. The original publication has also been updated.

## Figures and Tables

**Figure 1:**
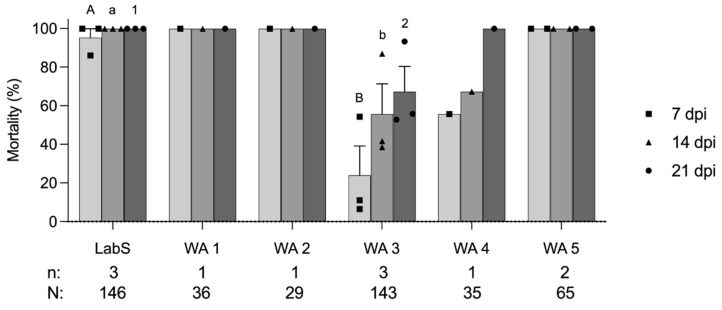
Mortality (mean ± standard error) at 7, 14, and 21 days post-infection (dpi) of six codling moth colonies LabS, WA1 to WA5 exposed to 6 × 10^4^ OB/mL GV-0001 (Cyd-X^®^). Each data point representing mortality at 7, 14, and 21 dpi was plotted as a square, triangle, and circle, respectively. Data were analyzed with *t*-test at *p* < 0.05. Different capital letters, lowercase letters, and numbers represent the significant differences of mortality at 7, 14, and 21 dpi, respectively. The number of replicates (n) and the total number of tested individuals (N) of each codling moth colony are shown below the chart.

**Figure 2:**
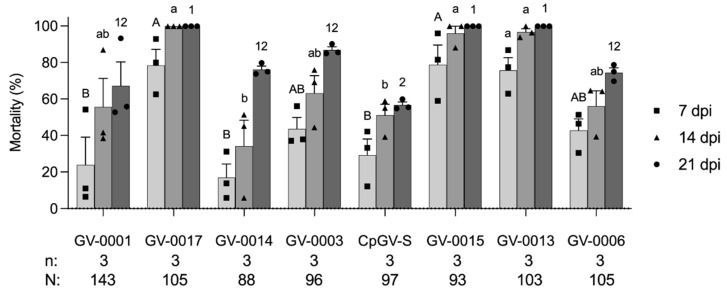
Mortality (mean ± standard error) of first instars of codling moth from WA3 colony exposed to eight CpGV formulations at a concentration of 6 × 10^4^ OBs/mL. Mortality was recorded at 7, 14, and 21 days post infection (dpi). Each data point representing the mortality at 7, 14, and 21 dpi was plotted as a square, triangle, and circle, respectively. Data were analyzed with one-way ANOVA followed by Tukey–Kramer HSD comparison at *p* < 0.05. Different capital letters, lowercase letters, and numbers represent the significant differences of mortality at 7, 14, and 21 dpi, respectively. All tested individuals (N) and replicates (n) are shown below the chart.
