# Supplementary material for: First Evidence of CpGV Resistance of Codling Moth in the USA
Source: Insects. 2022 Jun 10;13(6):533. doi: 10.3390/insects13060533 (PMC9225026; doi:10.3390/insects13060533)
Supplement: Supplementary file 1 [file insects-13-00533-s001.zip › insects-1623625-supplementary-12.2.pdf]

insects

MDPI

Supplementary Materials:

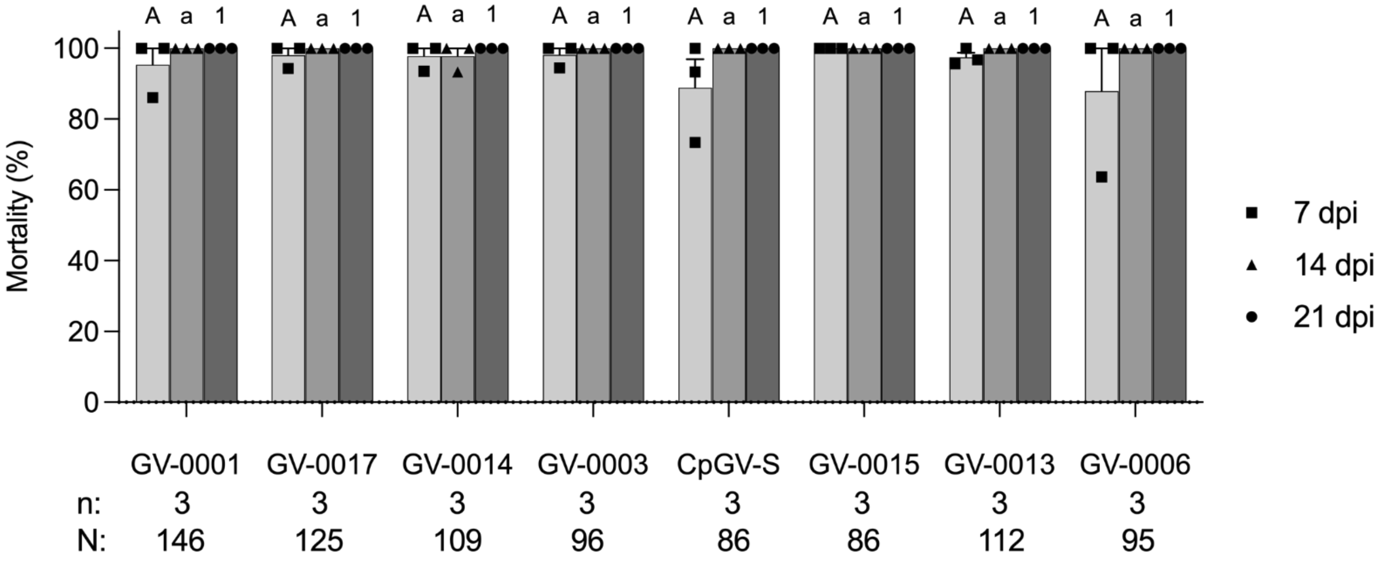

Figure S1. Susceptibility (mean ± standard error) of eight CpGV products to LabS colony. The mean mortality was Abbott corrected at 7, 14, and 21 days post-inoculation (dpi). Each data point representing the mortality at 7, 14, and 21 dpi was plotted as a square, triangle, and circle, respectively. Data were analyzed by one-way ANOVA followed by Tukey–Kramer HSD comparison at $p < 0.05$. Different capital letters, lowercase letters, and numbers represent the significant differences of mortality at 7, 14, and 21 dpi, respectively. All tested individuals (N) and replicates (n) are shown below the chart.
